# High Spatiotemporal Resolution ECoG Recording of Somatosensory Evoked Potentials with Flexible Micro-Electrode Arrays

**DOI:** 10.3389/fncir.2017.00020

**Published:** 2017-04-11

**Authors:** Taro Kaiju, Keiichi Doi, Masashi Yokota, Kei Watanabe, Masato Inoue, Hiroshi Ando, Kazutaka Takahashi, Fumiaki Yoshida, Masayuki Hirata, Takafumi Suzuki

**Affiliations:** ^1^Graduate School of Frontier Biosciences, Osaka UniversityOsaka, Japan; ^2^Center for Information and Neural Networks, National Institute of Information and Communications Technology and Osaka UniversityOsaka, Japan; ^3^Department of Organismal Biology and Anatomy, University of ChicagoChicago, IL, USA; ^4^Endowed Research Department of Clinical Neuroengineering, Global Center for Medical Engineering and Informatics, Osaka UniversityOsaka, Japan

**Keywords:** μECoG, somatosensory evoked potential, finger somatotopy, brain-machine interface, machine learning, sensory decoding

## Abstract

Electrocorticogram (ECoG) has great potential as a source signal, especially for clinical BMI. Until recently, ECoG electrodes were commonly used for identifying epileptogenic foci in clinical situations, and such electrodes were low-density and large. Increasing the number and density of recording channels could enable the collection of richer motor/sensory information, and may enhance the precision of decoding and increase opportunities for controlling external devices. Several reports have aimed to increase the number and density of channels. However, few studies have discussed the actual validity of high-density ECoG arrays. In this study, we developed novel high-density flexible ECoG arrays and conducted decoding analyses with monkey somatosensory evoked potentials (SEPs). Using MEMS technology, we made 96-channel Parylene electrode arrays with an inter-electrode distance of 700 μm and recording site area of 350 μm^2^. The arrays were mainly placed onto the finger representation area in the somatosensory cortex of the macaque, and partially inserted into the central sulcus. With electrical finger stimulation, we successfully recorded and visualized finger SEPs with a high spatiotemporal resolution. We conducted offline analyses in which the stimulated fingers and intensity were predicted from recorded SEPs using a support vector machine. We obtained the following results: (1) Very high accuracy (~98%) was achieved with just a short segment of data (~15 ms from stimulus onset). (2) High accuracy (~96%) was achieved even when only a single channel was used. This result indicated placement optimality for decoding. (3) Higher channel counts generally improved prediction accuracy, but the efficacy was small for predictions with feature vectors that included time-series information. These results suggest that ECoG signals with high spatiotemporal resolution could enable greater decoding precision or external device control.

## Introduction

The electrocorticogram (ECoG) has been proposed as a well-balanced source signal that may be especially applicable to clinical brain machine interfaces (BMI) because it is less invasive and has high signal fidelity compared with other techniques(Schalk and Leuthardt, 2011). Until recently, ECoG electrodes were generally used to identify epileptogenic foci in clinical situations, using large electrodes that typically had a diameter of ~4 mm and an inter-electrode distance of ~10 mm. However, a large number of recent studies have employed methods with higher electrode density, higher channel counts, and smaller electrodes (Rubehn et al., 2009; Ledochowitsch et al., 2011; Matsuo et al., 2011; Toda et al., 2011; Viventi et al., 2011; Escabí et al., 2014; Castagnola et al., 2015; Kellis et al., 2015; Khodagholy et al., 2015; Hotson et al., 2016). While high-density ECoG recording seems to improve BMI performance, for instance, by enhancing naturalistic control of prosthetic arms, few studies have directly demonstrated the efficacy of this technique. Recently, Wang and colleagues compared the efficacy of high-density ECoG arrays to that of conventional clinical arrays, based on the accuracy of finger movement classification (Wang et al., 2016). To our knowledge, their study produced some of the first practical evidence for the efficacy of high-density ECoG. However, the authors used high-density arrays with a comparatively large electrode, i.e., 64 channels, a diameter of 2 mm, and an inter-electrode distance of 4 mm.

Several reports have discussed the sizes and inter-electrode distances of ECoG electrodes (Freeman et al., 2000; Slutzky et al., 2010; Wodlinger et al., 2011; Muller et al., 2016). If the area covered by an electrode array is constant, then an increase in electrode density will enable the collection of brain activity at a higher spatial resolution, which could result in a larger and more comprehensive data set. Indeed, whether higher density electrodes are advantageous in all situations is not clear.

ECoG signals are both spatial and temporal, necessitating the use of a sampling theorem with a spatial domain. If the acquisition of signals with a maximum spatial frequency *f* is desired, it will be necessary to locate each sensor with a density greater than 2*f* after eliminating frequency components >*f* via filtering (in practice, pre-filtering is difficult in ECoG sampling). Otherwise, aliasing will occur and the signal will be distorted (Wolpaw and Wolpaw, 2012). According to this principle, increasing the spatial resolution of sampling will simply require an increase in sensor density.

However, in designing an actual electrode array, simply increasing the electrode density may not be beneficial. First, if the channels are located more closely to one another, the correlation and coherence of the signals will increase and the signals may become indistinguishable. This effect is more pronounced in lower frequency bands (Muller et al., 2016). Additionally, signal power decreases when the spatial frequency of a signal increases, and there may exist a threshold beyond which it becomes difficult to distinguish the signal from white noise in the spatial domain (Freeman et al., 2000). This limit (corner frequency) is thought to depend on electrode impedance, size, amplifier performance, and related factors. In addition, higher density electrode arrays will unavoidably have smaller individual electrode contacts, resulting in a higher electrode impedance and lower signal-to-noise ratio (SNR). For the reliable use of ECoG arrays in daily BMI applications, signals must be resistant to ambient noise, such as motion artifacts or electromagnetic interference. Another problem suggested by a modeling study is that smaller electrode contacts can sense only shallower neurons (Wodlinger et al., 2011). Fabrication cost and wiring challenges also exist. These factors must be considered when designing a high-density ECoG array to meet overall BMI system design objectives.

Previous studies have produced some theoretical indications regarding optimal inter-electrode intervals. Using spatial spectral analysis of ECoG signals, Freeman et al. reported that >1.25 mm was an optimal inter-electrode interval (Freeman et al., 2000). Slutzky et al also demonstrated the optimality of electrode contacts spaced in intervals ranging from 1.7 to 1.8 mm for human and 0.6 mm for rat brains. They used a finite element method that assumed a single dipole and subdural electrode placement. This difference between humans and rats can be largely accounted for by the distance between the electrodes and current source in the two different species (cortical thickness) (Slutzky et al., 2010).

The above-mentioned values are intrinsically dependent on the targeted cortical regions and frequency bands of interest. Nevertheless, these estimations are based on electrode arrays with a higher density than those used in the above-mentioned report by Wang. When assessing these theoretical investigations, we considered it valuable to investigate the efficacy of arrays with a much higher density, i.e., those with sub-millimeter intervals or actual areas of contact.

The organization of this paper is as follows: First, we introduce our new high-density flexible electrode array (inter-electrode distance: 700 μm, recording site: 350 μm square) and describe the successful recording and visualization of macaque finger SEPs with a high spatiotemporal resolution. Second, we evaluate the efficacy of high-density electrode arrays using machine learning analyses in which stimulated fingers and intensities were predicted from recorded SEP waveforms. Finally, we show that higher channel counts could improve prediction accuracy via channel subsampling and channel averaging analysis.

## Materials and methods

### Electrophysiology

#### Electrode fabrication

**Figure 1:**
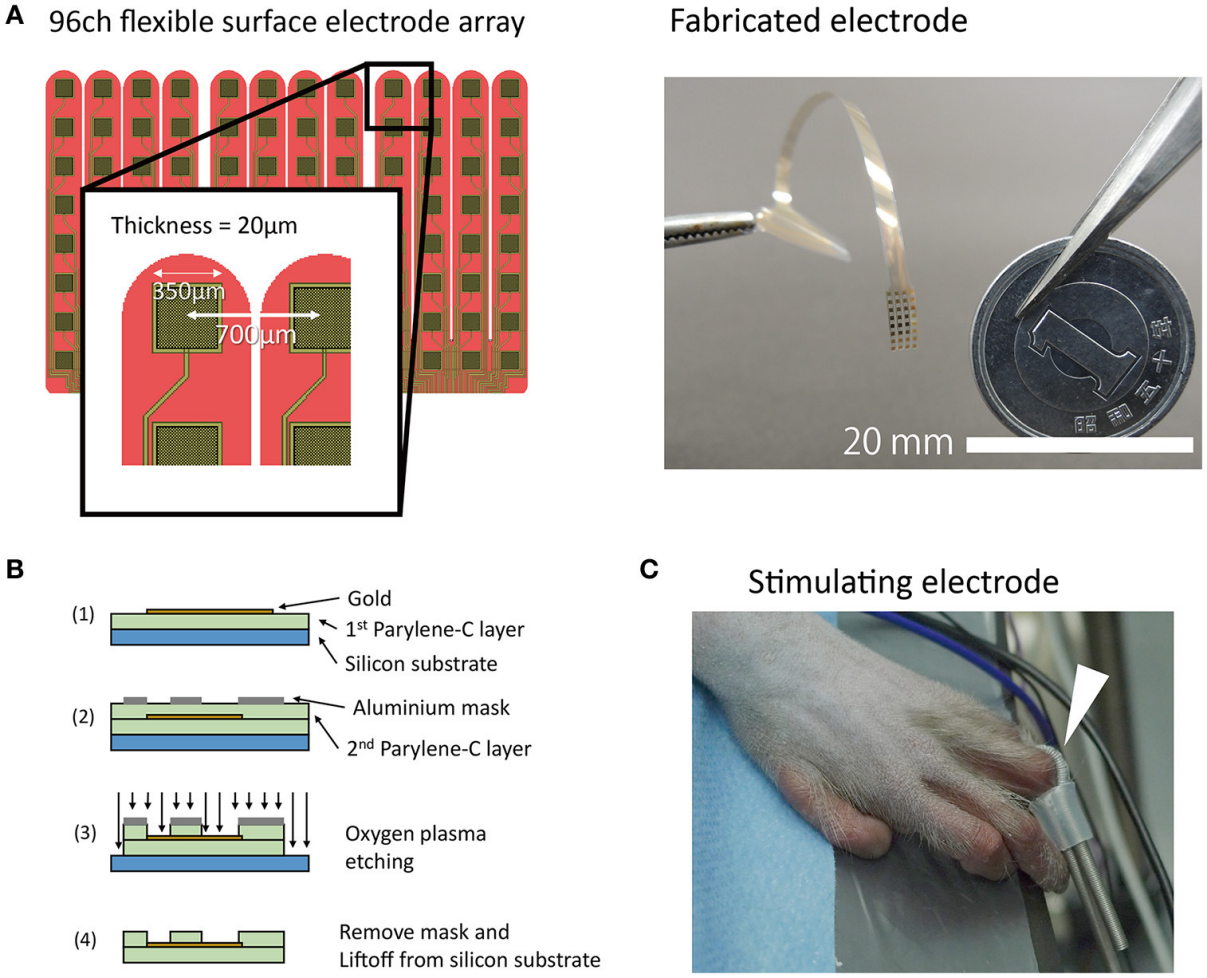
**Electrode fabrication and experimental paradigm. (A)** A schematic drawing and photograph of the electrode. Note that the tip of the electrode was separated like a comb. In the picture, the connecter portion is held by a pair of tweezers. **(B)** Schematic drawing of fabrication process. **(C)** A coil electrode for finger stimulation (white arrow). The coil electrode had a loop structure that was retained by a short silicone tube. The loop was wound around the monkey's finger.

We designed and fabricated a Parylene-C[poly(chloro-para-xylylene)]-based 96-channel (32 channels × 3 patches) flexible high-density electrode array (Figure 1A). The size of the array was set such that it would sufficiently cover the cortical representation of a hand. The inter-electrode distance was 700 μm, and the recording area of each channel was 350 μm square (0.12 mm^2^). Electrode fabrication was carried out using the same process described previously (Toda et al., 2011). Briefly, a Parylene layer (10 μm), patterned gold layer, and second Parylene layer (10 μm) were deposited one by one on a silicon substrate. The Parylene covering the gold contacts was eliminated via oxygen plasma etching (Figure 1B). A gold reference wire (φ0.76 mm, insulated without tip,) was attached externally. The mean electrode impedance was 11 ± 7.5 kOhm (mean ± S.D., at 1 kHz). Supplementary Figure 1 shows the frequency response curve for an electrode with the same design as those used in the current study.

#### Animal surgery

We used one female macaque monkey (7 y.o., 4.7 kg). Surgery was mainly performed by neurosurgeons. After durotomy, the surgeon referenced the anterolateral ending of the intraparietal sulcus to anatomically identify the finger representation area in the right postcentral gyrus. The central sulcus was opened according to a similar procedure described in a previous report (Matsuo et al., 2011). Following the removal of the arachnoid, the neighboring portion of the central sulcus (CS) was opened to the largest possible degree. The opened area had a depth of ~3 mm and length of 1 cm along the CS. Each electrode patch was placed onto the brain surface such that a portion of each patch rested inside the central sulcus (Supplementary Figure 2). A piece of saline-dipped gauze was placed onto the electrodes to stabilize the contact between the arrays and the cortical surface. A ground electrode was connected to anchor screws inserted into the cranial bone. A reference electrode was placed in subdural space. We conducted an acute recording session and, after data collection, removed the electrodes. Following data collection, an additional electrode implantation was performed as part of another study and the craniotomy was closed. All experimental protocols were in accordance with the Animal Research guidelines of Osaka University Graduate School of Frontier Biosciences. This study was approved by the animal experiment committee at Osaka University Graduate School of Frontier Biosciences.

#### Data collection

SEPs were recorded after administration of propofol, fentanyl, and isoflurane. Unfortunately, the detailed anesthesia records were lost. The approximate dosage of each drug was 4.25–8.5 mg·kg^−1^·h^−1^, 10.6 μg·kg^−1^·h^−1^, and 0–2%, respectively. Spontaneous breathing was maintained during the recording session. We observed no evidence of abnormal waveforms or suppression in the background ECoG activity. We used the electrical stimulator NS-101 (Unique Medical, Tokyo, Japan) for stimulation of fingers. A coiled stimulating electrode covered with conductive paste was placed between the proximal and distal interphalangeal joints of the left hand (Figure 1C). As a return electrode, another coil electrode was wound around the left forearm of the monkey. We used a single cathodal monophasic pulse (width 0.2 ms) and fingers were stimulated at two different intensities (1 and 4 mA). The stimulation interval was set at 305 ms. The stimuli in each condition (finger type and intensity) were continuously presented for a total of ~200 trials. The timing information for the stimulation triggers and neural signals was recorded using a neural signal amplifier RZ2 Bioamp (Tucker-Davis Technologies, Alachua Fl., USA). Data acquisition was performed at a sampling frequency of 6,103.52 Hz. See Supplementary Material for details about the stimuli presentation in each condition and examples of recordings that were excluded from analysis (Supplementary Table 1). In one condition (D2, 4 mA), the time data for the stimulation trigger and stimulus artifact were inconsistent. In this condition, trigger timing was adjusted manually by referencing the timing of stimulation artifacts.

### Data analysis

Data processing was carried out using MATLAB 2015b and 2016a (Mathworks, Natick MA, USA).

#### Common preprocessing

**Figure 2:**
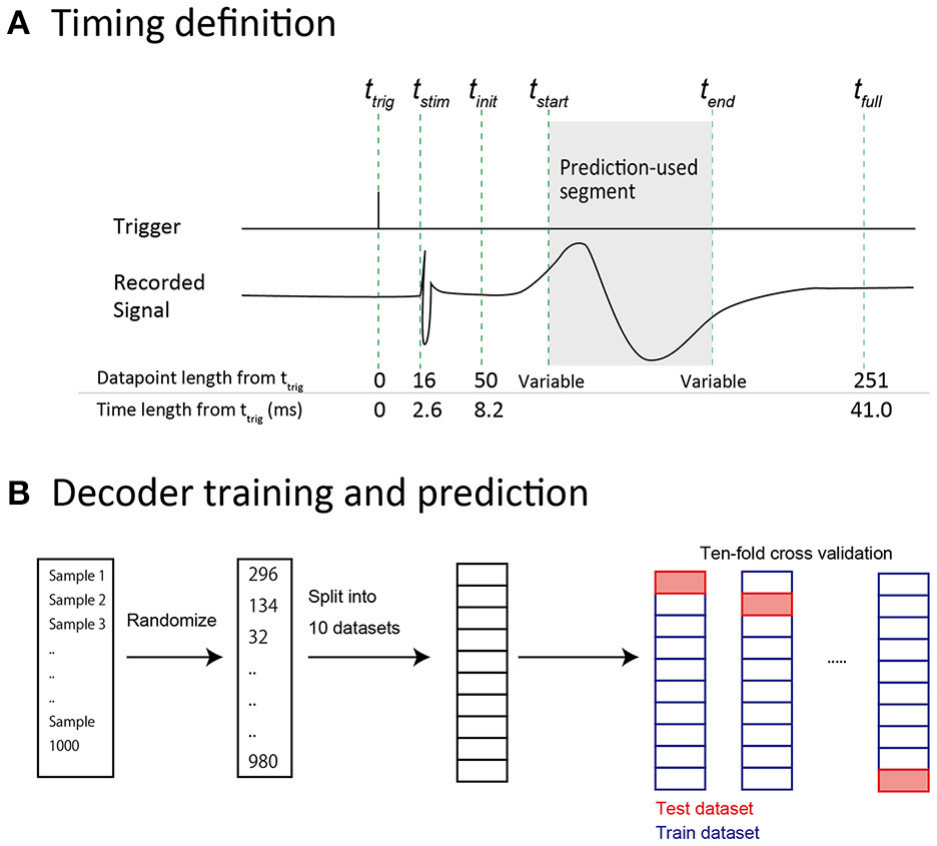
**Data preparation for predictive analysis. (A)** Time-point definitions of prediction samples. *t*_*trig*_: The time at which the stimulus trigger was generated by the recording equipment. *t*_*stim*_: The time at which the stimulus artifact was observed. The actual stimulus was considered to be delivered at this time. *t*_*init*_: The time at which 50 data points had elapsed from *t*_*trig*_. In predictive analysis, the data were acquired after this time point to prevent contamination by stimulus artifact. *t*_*start*_: The start time of the data segment that was included in a feature vector. *t*_*end*_: The end time of the data segment that was included in a feature vector. Note that *t*_*start*_ and *t*_*end*_ were variable. *t*_*full*_: The time point at which 250 data points had elapsed from *t*_*trig*_. We defined this (*t*_*trig*_−*t*_*full*_) length as “full length” in the prediction analysis. **(B)** Decoder training and prediction. All 1,000 samples were shuffled and split into 10 datasets. Accuracy was calculated using 10-fold cross validation. Each dataset (containing 100 samples) was used for prediction by a decoder, which was trained by the other 9 datasets (containing a total of 900 samples).

The raw signal for each condition was expressed as a 2-dimensional (2-D) matrix with 96 (channels) × N (data points). The stimulation trigger timing (t_*trig*_, Figure 2A) for each condition was also saved as a vector with 1 × N (data points). Two channels out of 96 showed noisy waveforms instead of an evoked potential, indicating a technical problem. These two broken channels were eliminated from analysis unless otherwise stated. The raw signal was re-referenced using common average referencing. Baselines were corrected for each sample by subtracting the mean voltage during a 98 ms period prior to t_*trig*_. Next, the data were formatted for SEP analysis and prediction analysis. The onset of *t*_*stim*_ (Figure 2A) was delayed by about 2.6 ms from *t*_*trig*_. This *t*_*trig*_-*t*_*stim*_ relationship was consistent in all conditions.

#### SEP analysis

**Figure 3:**
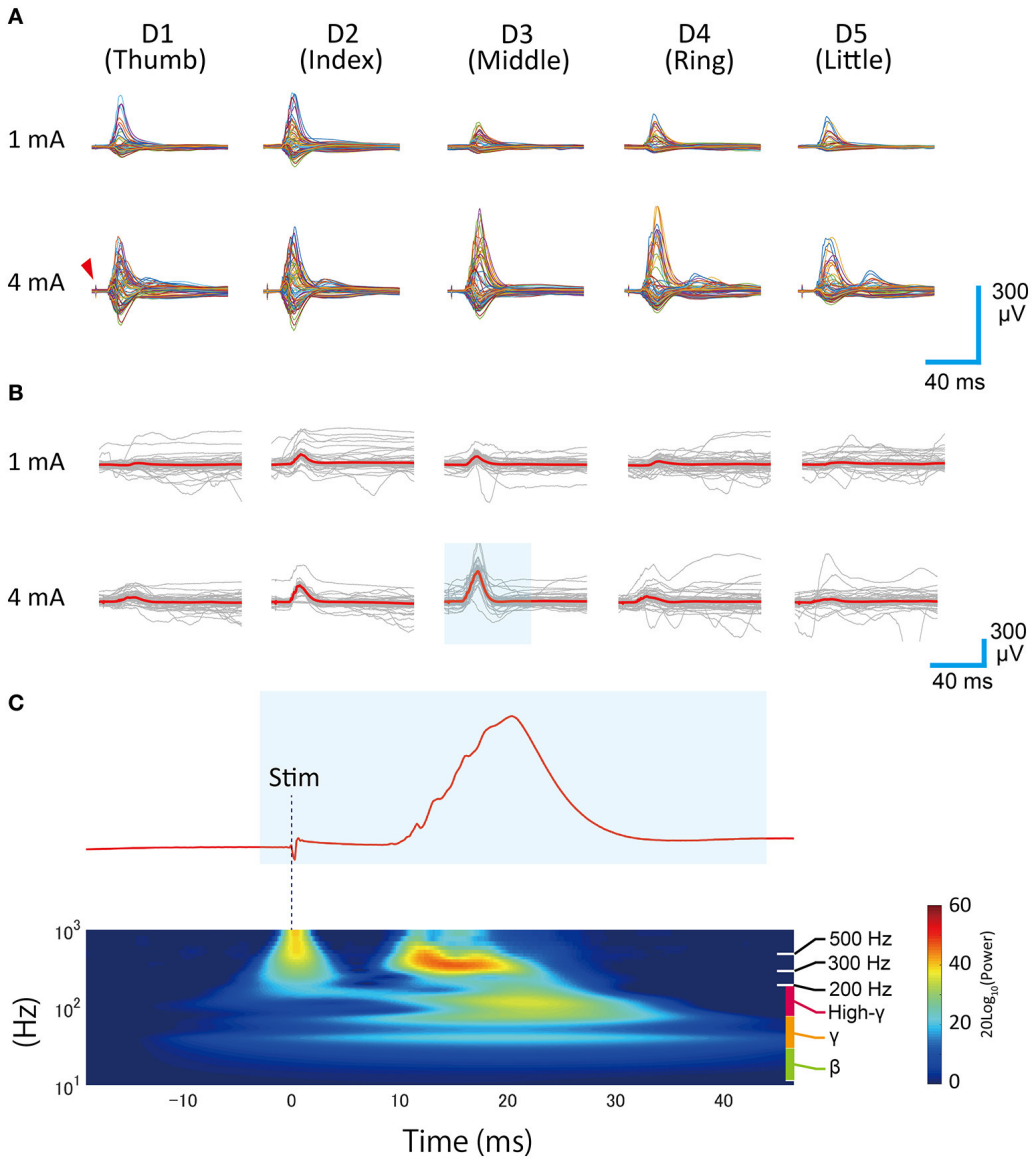
**Averaged SEP waveforms. (A)** Each row shows the different stimulation intensities and each column shows the stimulated finger type. Each single trace represents the time series data for one channel. A sharp biphasic wave appearing at the leading position (~3 ms) in each trace shows the direct artifact of electrical stimulation (red arrow). A slow positive/negative wave appearing around 20 ms after the stimulation trigger represents the short-latency somatosensory evoked potential. **(B)** Overlay plots of single stimulation responses, shown as gray traces. The red traces indicate the mean. These data were obtained from the representative channel (Ch 40). **(C)** Wavelet analysis of evoked response (representative data, 4 mA, D3). The area shaded in blue corresponds to the shaded area in **(B)**.

Extracted and formatted waveforms were averaged to obtain averaged SEP waveforms (Figures 3A,B). Because the recording sessions were manually orchestrated, the recording periods were slightly different among each recording condition (finger type and intensity). As a result, the number used for averaging varied among the recording conditions. Actual counts are shown in Table 1.

**Table 1:** **Average sample counts in each stimulation condition**.

**Condition**	**D1 1 mA**	**D2 1 mA**	**D3 1 mA**	**D4 1 mA**	**D5 1 mA**	**D1 4 mA**	**D2 4 mA**	**D3 4 mA**	**D4 4 mA**	**D5 4 mA**
Averaging counts	212	221	188	221	221	238	218	211	229	217

**Figure 4:**
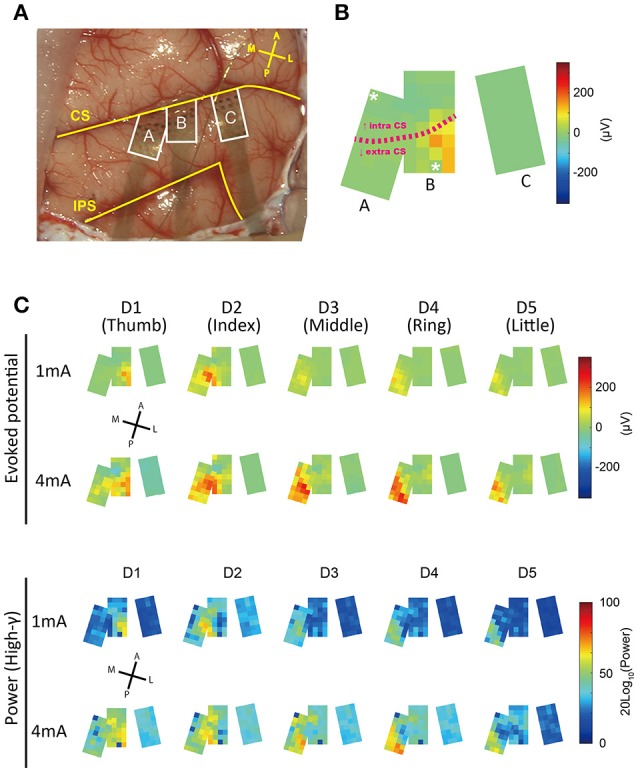
**Spatial patterns of responses evoked by finger stimulation. (A)** Operative view of electrode placement. The three areas surrounded by the white line represent each electrode patch. Patches A and B were partially overlapped. Each patch was flexible and curved along the brain surface. A section of each patch was inserted into the central sulcus. Landmarks and orientations: CS, central sulcus; IPS, intraparietal sulcus; A, anterior; P, posterior; M, medial; L, lateral. **(B)** Averaged surface potential distribution (1 mA, D1) at 18 ms after t_*stim*_. Characters assigned to each patch correspond to characters in **(A)**. The two channels with asterisks were broken. The pink dotted line indicates the approximate position of the central sulcus. The CS position in patch C was unknown. **(C)** Surface distributions of evoked responses.

Wavelet analysis (Figure 3C) was carried out using the cwtft function in MATLAB with the Morlet wavelet. The results were normalized with respect to the pre-stimulus period. A surface map of evoked responses (Figure 4) was made from the averaged SEP waveforms by converting the voltage values into pseudo color. A surface map of high-gamma power distribution was made by calculating the mean power during the 33 ms period following *t*_*init*_ (Figure 2) using the bandpower function in MATLAB. The supplemental videos were made by collecting all of the surface maps (evoked response map, 98 ms period after *t*_*trig*_, 600 frames) with a frame rate of 30 frames/s. Supplementary Videos 1 (1 mA), 2 (4 mA) show the responses obtained in the different stimulation conditions.

##### Center-of-mass trajectory of peak response

**Figure 5:**
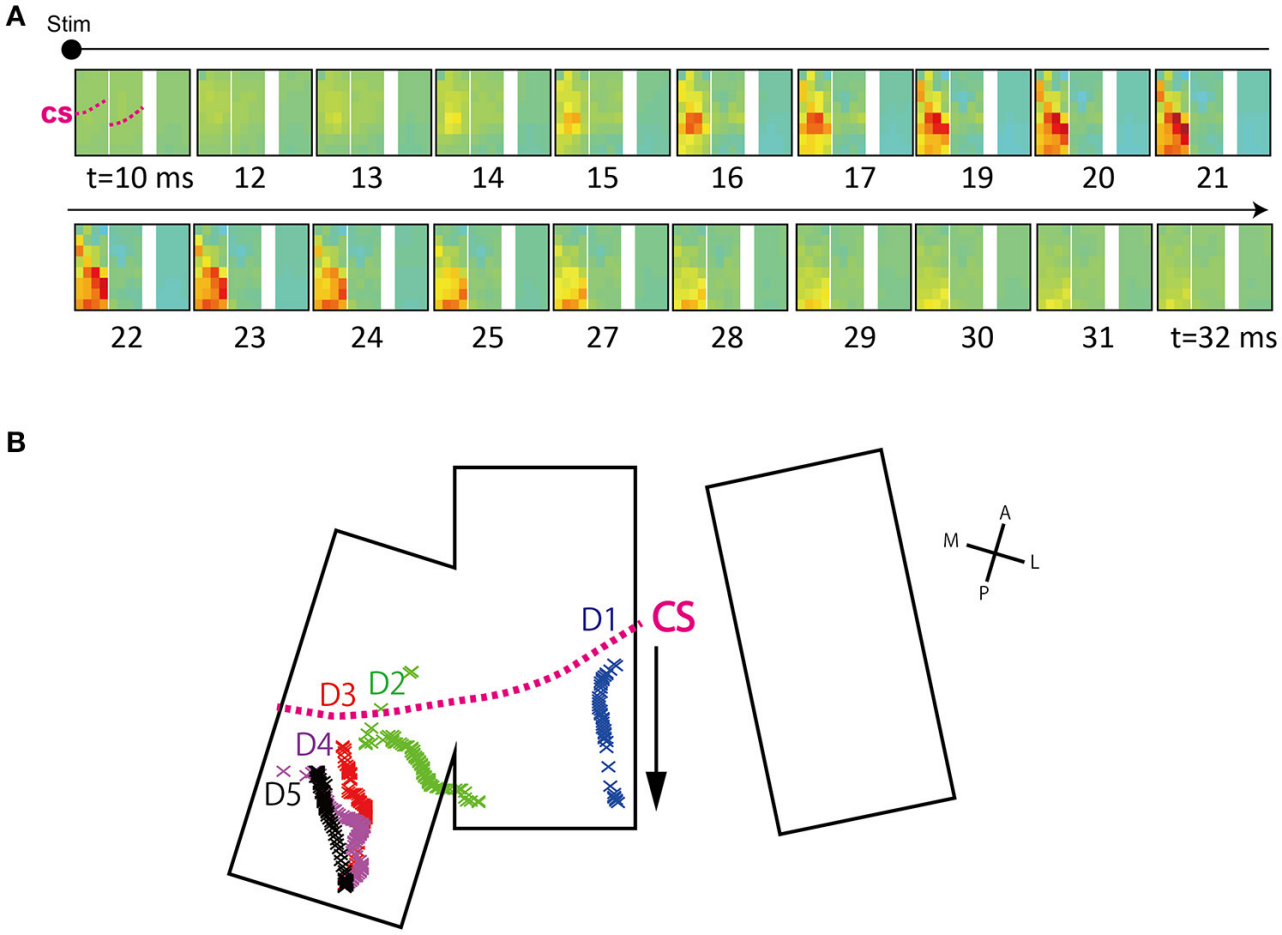
**Time course of surface potential distribution. (A)** Sequential images show the time course of surface potential evolution. Representative data are shown (D3, 4 mA). The timing at which stimulation artifact appeared (*t*_*stim*_) was set to *t* = 0 and images are displayed from 10 ms after *t*_*stim*_. Electrode arrays are shown in re-aligned form (From left, patch A, B, and C). Arrays actually have inter-spacing, as shown in Figure 4A. The displayed time was rounded off to the nearest integer. The pink dotted line labeled CS in the first frame represents the approximate central sulcus line. **(B)** Center-of-gravity (COG) trajectory of peak response area. Calculated COGs at each time point are indicated by cross marks. As shown by the arrow, COGs tended to move from the anterior to posterior direction. The data shown was obtained in the 4 mA condition. The CS line shows the approximate position of the CS. D1, Thumb; D2, Index; D3, Middle; D4, Ring; D5, Little.

Trajectories of peak responses (Figure 5B) were calculated as follows: As the electrode patches were not regularly aligned, spatial interpolation was conducted first. By referencing the patches to an intraoperative picture, each patch was re-located on an 800 × 513 2-D matrix. Although the actual electrodes were curved along the brain surface and one edge was positioned in the sulcus, the curved areas were visually manually positioned to approximate the position in the planar 2-D matrix area. Next, for each time *t*, the voltage value of each channel was assigned to the corresponding position on the 2-D matrix. As a result, voltage values for each channel were sparsely re-located on the 2-D matrix. The 2-D matrices were interpolated using the scatteredInterpolant function in MATLAB (parameters: natural, no extrapolation was conducted). Two broken channels and one overlaying channel were excluded from the interpolation.

Using this interpolated voltage map, we calculated the center-of-gravity (COG) of the peak response at each time *t*. Calculations were done using the equation below (modified from Reimer et al., 2011). Note that calculations were done only for areas with voltages above +150 μV.

$$\begin{array}{l} COG{\left(t\right)}_{x}=\frac{\sum_{j}x_{j}\;\cdot \text{ i}ECoG{\left(t\right)}_{j}}{\sum_{j}iECoG{\left(t\right)}_{j}},\\ COG{\left(t\right)}_{y}=\frac{\sum_{j}y_{j}\;\cdot \text{ i}ECoG{\left(t\right)}_{j}}{\sum_{j}iECoG{\left(t\right)}_{j}},\\ \;j=\{j\;|\;iECoG{\left(t\right)}_{j}\;\geq \;\;150(\mu \text{V})\}. \end{array}$$

Here, an averaged ECoG voltage, which is interpolated at time *t* and position *j*, is shown as *iECoG*(*t*)_*j*_. A coordinate of position *j* is shown as (*x*_*j*_, *y*_*j*_). The trajectory map is obtained by sequentially calculating (*COG*(*t*)_*x*_, *COG*(*t*)_*y*_).

We investigated threshold levels to produce comparable trajectory lengths for each finger. We adopted a threshold of 150 μV. The differences in inter-channel signal noise levels were small (see Results) such that the constant threshold was deemed to be sufficient.

##### Noise level evaluation

For noise level evaluation, we calculated the root mean square value of the rest-state (inter-stimulus) ECoG voltage using a 98-ms segment starting 100 ms before stimulation onset (*t*_*stim*_). This segment had no apparent evoked response. Before calculation, the mean of each extracted segment was set to zero.

#### Finger and intensity prediction analysis

We performed multi-class classification analyses (5 finger types × 2 intensities = 10 conditions). We used SEP waveforms (time series of voltage values, non-spectral data) as feature vectors. Waveform extraction was conducted using the same method described in the common preprocessing section. We defined this extracted waveform as a “full-length sample.” A full-length sample had a length of 200 data points (*t*_*init*_–*t*_*full*_; about 33 ms, Figure 2A). In some of the analyses described below, we used segments of these full-length samples for the purpose of manipulating the length of time-series information in feature vectors. We defined the start position of cutting as *t*_*start*_ and the end position as *t*_*end*_ (Figure 2A), such that a segment that was included as a feature vector was defined from *t*_*start*_–*t*_*end*_. If an entire full-length sample was used in a prediction, *t*_*start*_ was set to *t*_*init*_, and *t*_*end*_ was set to *t*_*full*_.

In all prediction analyses, we used only 100 samples from each condition (samples recorded from the 1st to the 100th stimulation) to match the number of different class samples. Hence, we collected a total of 1,000 samples (10 conditions × 100 stimuli). Each sample was converted to a z-score using the all-sample mean and standard deviation.

We adopted a linear-kernel support vector machine as a machine learning algorithm, which was fast and easily tunable. Before calculating predictions, it was necessary to establish a hyperparameter, termed C (penalty parameter). We plotted and investigated the relationships between C and accuracy for each analysis. A parameter space ranging from 10^−10^ to 10^2^ was divided into 20 points in logarithmic space. In each analysis, a single globally effective parameter was visually located and adopted in the prediction. As a result, in the analysis of spatial resampling without time-series data (**Figure 8B**), C = 10^−1^ was used according to prior investigations (Supplementary Figure 3). In all other analyses, C = 10^0^ was used (Supplementary Figures 4–6).

The prediction results were verified with 10-fold cross validation (Figure 2B): A test data set was split into 10 subsets. Prediction was performed for each subset (including 100 samples, 10% of all samples) using a decoder trained with the other 9 subsets. Accuracy was defined by (the number of correct predictions)/(the number of all predictions). Decoder training and the prediction algorithm were implemented with Python and the Scikit-learn machine learning library.

##### Temporal truncation analysis

To investigate the importance of temporal information for prediction, we limited the temporal information input of the data. We set *t*_*start*_ = *t*_*init*_. As an initial sample, we obtained a single data point × 94 channel length vector (*t*_*start*_–*t*_*end*_ had a length of one data point: about 0.16 ms). Hereafter, input samples were stepwise elongated by one data point and each sample was evaluated with 10-fold cross validation.

##### Spatial subsampling analysis

To investigate the importance of spatial information, we used the prediction method and limited the spatial input of the data.

In time-series data prediction (**Figure 8A**), we set *t*_*start*_ = *t*_*init*_. *N* channels were randomly selected from all 94 channels and a sample with a length of *T* data points was obtained for each channel. We prepared feature vectors with size *N* (channels) × *T* (data points) as one training/test sample. The number of channels *N* was varied from 1 to 91 with a step size of 5. For each *N*, the data point length T was varied from 1 to 196 with a step size of 5. For each combination of *N* and *T*, we calculated and plotted the mean prediction accuracy, evaluated by 10-fold cross validation.

For non-time-series data prediction (**Figure 8B**), a single time point in the ECoG signal (voltage) was used so that *t*_*start*_–*t*_*end*_ had a length of one data point (about 0.16 ms). *t*_*start*_ was initially set to *t*_*init*_ and varied until *t*_*full*_ with a step size of 5 data points. Feature vectors with size *N* (channels) × 1 (data point) were prepared as one training/test sample.

##### Merged channel analysis

We used limited time-series information in this analysis because we were not able to see the channel-count effects when using full-length samples. To ensure that we used waveform segments with predictive value, we set *t*_*start*_ to a point 20 ms from *t*_*stim*_ (86 data points from *t*_*stim*_) according to the results shown in **Figure 8B**. The time-series length (*t*_*start*_–*t*_*end*_) was set to 10 data points, i.e., about 1.6 ms.

Channel groups are indicated by red rectangles in **Figure 9A**. For each channel group, the signals from all channels included in the group were averaged and made into one signal. These signals could be thought of as having been recorded from “virtual large channels” comprising all of the channels in each group. Using these reconstructed signals, we conducted prediction analysis. The two broken channels were eliminated from this analysis.

## Results

### Spatiotemporal dynamics of finger SEP

We successfully recorded SEPs from our electrode arrays during electrical finger stimulation (Figure 3A). Large-amplitude positive waves were observed about 20 ms after stimulation onset. In some channels, we could see not only positive waves but also shallow negative waves or shallow positive/negative wave complexes.

In a monkey SEP study, McCarthy and colleagues reported the presence of “P10–N20” on the anterior surface of the central sulcus (CS), “N10–P20” on the posterior surface of the CS, and “P12–N25” in the vicinity of the CS. These waveforms are thought to be equivalent to the human “P20–N30”, “N20–P30,” and “P25–N35” (McCarthy et al., 1991). In this finger-stimulating SEP study, we found waveforms corresponding to the monkey “P12–N25” or “N10–P20” in some channels, which confirmed the successful recording of short latency somatosensory evoked potentials.

Qualitative investigation of wavelet analyses suggested that the observed SEPs consisted of three main components: First, a slow component with a main frequency around 40–50 Hz, second, a fast component, which ranged from 80 to 200 Hz (high-gamma), and third, a very fast component, which ranged from 300 to 800 Hz (Figure 3C). The first slow and the second fast components had almost the same spatiotemporal properties and emerged ~20 ms from stimulation onset in trials where the main SEP waves were observed.

For the purpose of visualization, we made surface potential distribution maps; color maps in which voltages at 18 ms after stimulation onset (*t*_*stim*_) were assigned pseudo colors (Figure 4C). We observed a progression in the peak position of SEPs from lateral to medial as the stimulated finger advanced from D1 (thumb) to D5 (little finger). This relationship between stimulated fingers and the spatial specificity of peak timing corresponded to known somatosensory somatotopy (Nelson et al., 1980; Pons et al., 1987). In addition, the area for which the high-gamma band exhibited the highest power was similar to the area where the raw potentials showed the highest amplitude.

Because we used a high-density electrode array with a high number of channels, we were able to visualize the detailed spatiotemporal dynamics of evoked potentials. We made a figure (Figure 5A) and movies (Supplementary Videos 1, 2) demonstrating the temporal evolution of activity. We observed that channels with well-defined peaks moved along the anterior-posterior axis. This movement was confirmed by a COG analysis of the peak response area (Figure 5B).

We calculated voltage fluctuations in the rest state to check the signal quality. For non-averaged data, the root-mean-square (r.m.s.) of the rest ECoG voltage was 18 ± 6.0 μV (inter-channel mean ± S.D.). For the averaged data, the r.m.s. was 1.9 ± 0.53 μV. As seen in Figure 3, recorded evoked responses were several hundred microvolts in size. Thus, high SNR was achieved and channel impedances were considered to be nearly equal.

We checked the array placements for any drifting, and found no evidence of movement. Specifically, we compared the pre-recording (D3, 4 mA) data acquired before recording the analyzed dataset with the last recording of the experiment (D3, 4 mA), and found no differences in the locations of channels with peaks (data not shown).

### Prediction of stimulated fingers and intensity using machine learning

#### Prediction results

**Figure 6:**
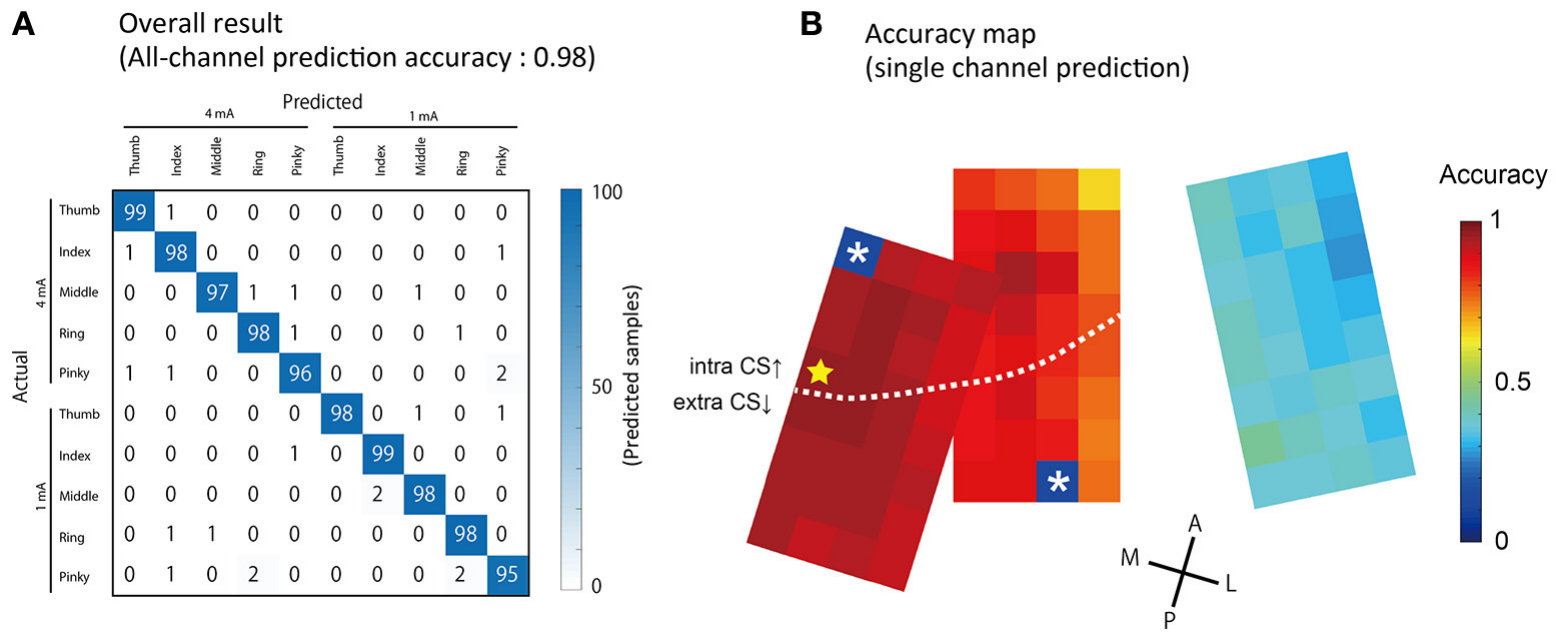
**Prediction results of stimulated finger type and intensity. (A)** The results of the prediction using all 94 channels to classify five finger types × two intensities = 10 conditions. Columns show actual (true) conditions and rows show predicted conditions. Each number in a cell corresponds to the number of the predicted samples. **(B)** Accuracy obtained from single-channel predictions are showed by pseudo colors. The yellow star indicates the channel with maximum accuracy (Ch 41, 0.96 ± 0.01, mean ± S.D.). The two asterisks indicate broken channels.

Stimulated fingers and intensity were successfully predicted by ECoG signals acquired from a single stimulation event (non-averaged SEP waveforms). Using all 94 channels, we conducted 10-condition classification (5 finger types × 2 stimulation intensity) with an accuracy of 0.98 ± 0.008 (mean ± S.D., Figure 6A). To compare this performance against chance levels, we also made a prediction using a dataset where the condition labels had been randomly shuffled, and obtained an accuracy of 0.098 ± 0.026.

We also performed predictions using a single channel; the accuracy is shown in Figure 6B. Channels with a higher accuracy were often observed in the medial area, in which large amplitude SEPs were recorded. The channel with the strongest predictive power, located in the most medial section of the arrays, had an accuracy of 0.97 ± 0.017 (Figure 6B, indicated by the star).

#### Temporal truncation analysis

**Figure 7:**
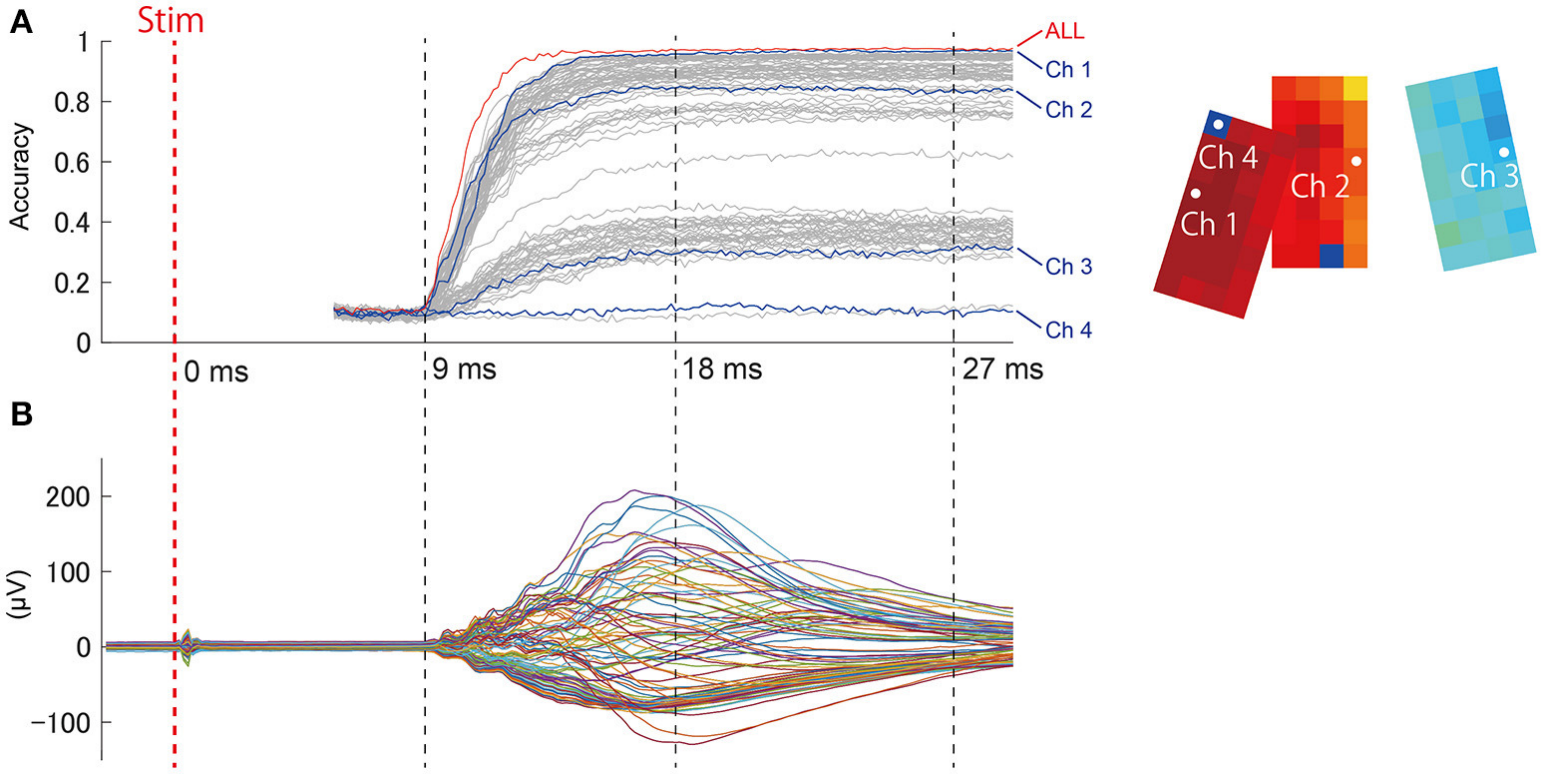
**(A)** (Left) Accuracy at *t* ms from the stimulation onset was calculated from the ECoG signal obtained prior to *t* ms. The red trace (“ALL”) shows the prediction accuracy obtained from all channels together (94 channels), and the other plot shows single-channel predictions. Data sections from the stimulation trigger to 5.2 ms afterwards might include the effects of direct artifact from electrical stimulation, and were thus excluded from prediction samples. (Right) The channel locations are shown on the accuracy map (same as Figure 6B). **(B)** For reference, the representative SEP waveform (D1, 4 mA) is displayed for the same time points as the accuracy plot.

As shown in Figure 5, responses evoked by stimuli were not constant, but exhibited spatial-temporal dynamics. Presumably, these dynamics could affect the prediction results. We were interested in determining what signal duration during stimulus presentation would be necessary for accurate prediction, as the latency between stimulus and prediction is an important factor in BMI applications, particularly perceptive BMI. We performed step-wise elongation of the time-series data and plotted a time course of accuracy (Figure 7A). Accuracy immediately reached a maximal value ~15 ms after stimulation onset and remained constant at this maximal value afterwards. We found that we could achieve accurate prediction using a relatively early and short signal duration from the dataset.

During time intervals without any evoked responses (from “Stim” to 9 ms, Figure 7B), prediction accuracy was maintained within chance levels (Accuracy = 0.1). This suggests that the contribution of stimulation artifact was sufficiently eliminated. This also ensured that the evoked response waveform itself had contributed to the prediction.

#### Channel counts and prediction accuracy: spatial subsampling analysis

**Figure 8:**
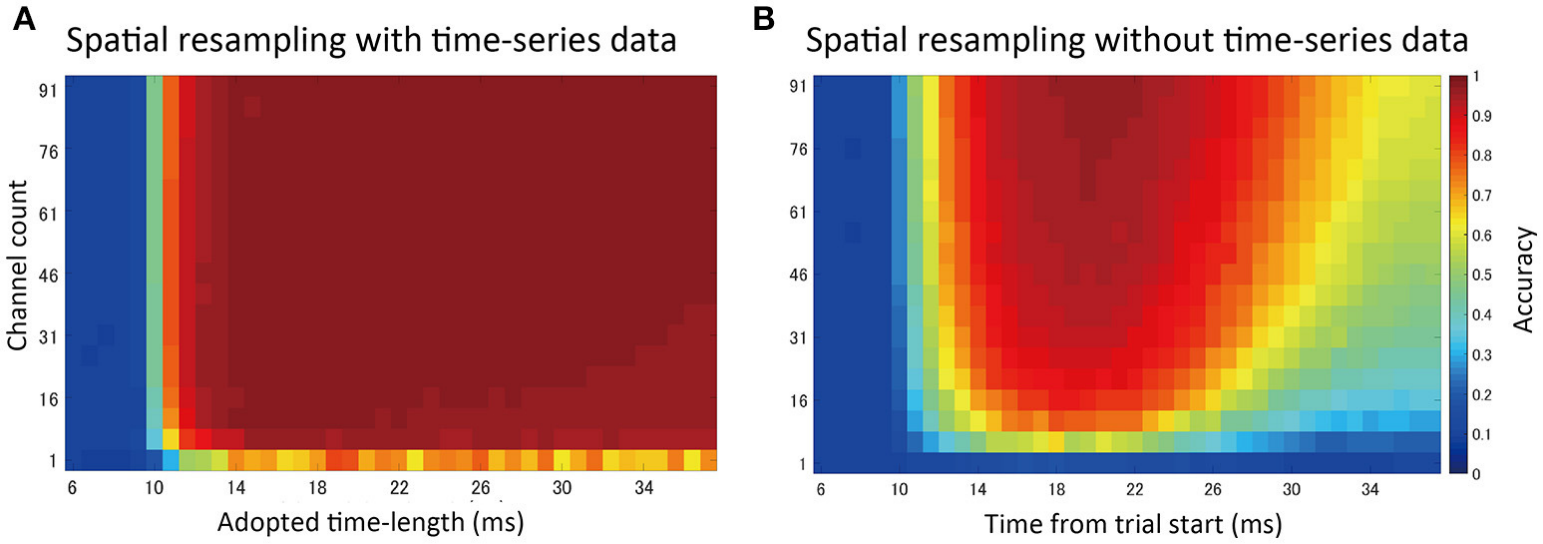
**Spatial subsampling analysis. (A)** Result of random spatial (channel) subsampling with time-series information in feature vectors. The vertical axis shows the number of sampled channels. The horizontal axis indicates the time-series length used in the prediction (*t* = 0 indicates stimulation timing, *t*_*stim*_). Sampling was repeated 30 times and the mean accuracy was plotted. **(B)** Result without time-series data. A single ECoG signal (voltage value) at one time point was used for prediction. The horizontal axis shows different values corresponding to that time point.

By randomly selecting channels to be included in the feature vectors (spatial subsampling), we investigated the effects of changing the density and the number of channels on prediction accuracy (Figure 8).

Generally, higher channel counts led to higher prediction accuracy. When time-series information was included in the feature vectors (Figure 8A), only a small number of channels were needed to achieve high accuracy, up to the point where increasing the number of channels further had a limited effect. Without time-series information (Figure 8B, voltage values at a single time point were used in feature vectors), accuracy varied depending on the selected time points. Additionally, higher accuracy was achieved at the time points with higher SEP amplitudes.

#### Channel counts and prediction accuracy: merged channel analysis

**Figure 9:**
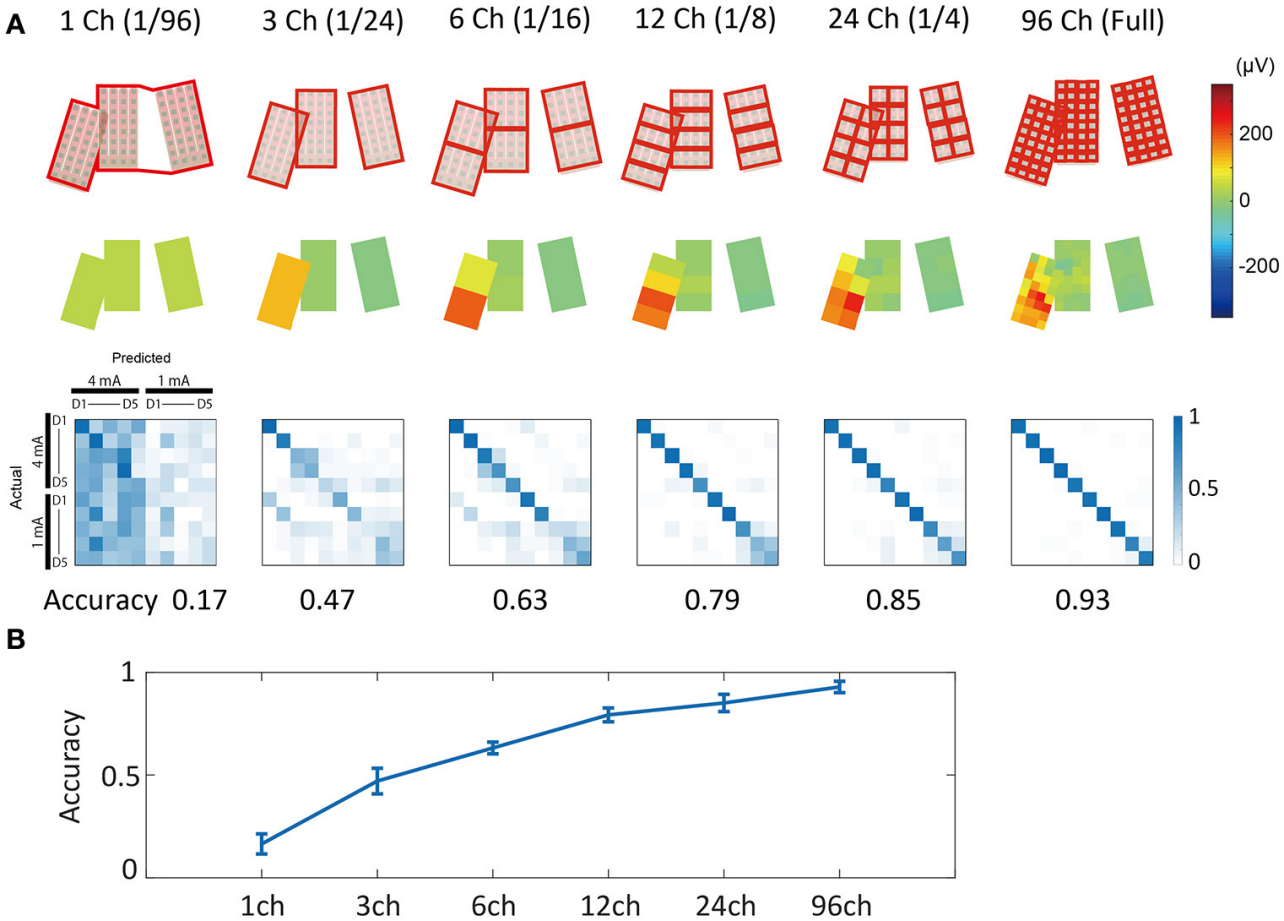
**Merged channel analysis. (A)** (Upper) Channel group definition. Channel signals included in the same red rectangle were averaged and made into a single signal. (Middle) Example of averaged surface potential distribution. Potentials at 18 ms after stimulation (4 mA, D3) are shown. (Lower) Confusion matrices of prediction results. Numbers under the matrices indicate prediction accuracy. Note that for this analysis, we used 1.6 ms of limited time-series information. **(B)** Channel count vs. accuracy plot. Increasing channel counts improved prediction accuracy. Error bars indicate mean ± S.D. of 10-fold cross validation results.

An averaged potential between two neighboring channels is considered to reflect the potential that the two electrically connected channels generate (e.g., average reference in EEG). Using this inter-channel averaging method, we investigated the prediction accuracy of the “virtual large channel” (Figure 9). As a result, we found that increasing the number of channels improved prediction accuracy. This improvement was larger in the 1 mA compared with the 4 mA condition, and also larger in the D3, D4, and D5 conditions compared with the D1 and D2 conditions. In this analysis, we limited the time-series information input to 1.6 ms because the full-length data points increased accuracy to a nearly maximal plateau level in most conditions. The input time-series was cut and extracted from the time point 20 ms after stimulation onset.

## Discussion

### Finger SEP recording with high-density arrays

In this study, higher density electrode arrays and other high performance recording equipment enabled us to perform SEP recording that was spatiotemporally precise and obtain finger somatotopy that was clearly delineated. The obtained somatotopy exhibited a gradient that was lateral to the medial axis as stimulated fingers advanced from D1 to D5. This result was consistent with monkey multi-unit studies with penetrating microelectrodes (Nelson et al., 1980; Pons et al., 1987), a human study with fMRI (Kolasinski et al., 2016), and monkey studies with optical imaging of intrinsic signals (Chen et al., 2001; Shoham and Grinvald, 2001).

A recent study reported a method for delineating finger somatotopy using high-density ECoG, mainly for the purposes of surgical planning, such as epileptogenic focus or tumor resection surgery (Prueckl et al., 2015; Wahnoun et al., 2015). Our use of novel electrode arrays with higher density and higher channel counts made it possible for us to obtain more detailed spatiotemporal delineation. Thus, the efficacy of high-density ECoG was re-confirmed not only for BMI applications, but also for clinical functional mapping.

### Effect of high-density electrode on finger prediction

Spatial sampling analysis (Figure 8) and merged channel analysis (Figure 9) showed that the use of a high-density ECoG electrode led to relatively high decoding accuracy.

According to the trajectory map (Figure 5B), D3, D4, and D5 were only separated from one another by approximately one channel interval (700 μm). Our high-density electrode array could successfully delineate these slight differences, thus leading to high prediction accuracy. However, some portions of the trajectories overlapped and could not be separated. Our map was generated exclusively from interpolated data obtained from an electrode with a channel interval of 700 μm. Thus, there is much potential for improvement in terms of trajectory separation and prediction accuracy when using electrode arrays with a higher density than those used in this study.

In this study, we were unable to clearly determine the efficacy of high-density electrodes for making predictions when time-series information is included in the feature vectors (Figure 8A). It is possible that time-series information could compensate for the loss of spatial information in our test paradigm. This may be the case with respect to the propagation dynamics of cortical responses. The response to a stimulus is not localized, but moves within a comparatively large area in a time-dependent manner. Thus, we could detect the response pattern even if the sensor was not precisely located to capture the initial portion of the response. Conversely, when using limited time-series information, a high channel count could recover the loss of time-series information to some extent. Thus, this relationship may be bidirectional.

With regard to daily-life applications of BMI, limitations to equipment design, such as sampling frequency limits or low SNR, are likely. Our data support the important possibility that increased channel counts and density can compensate for the loss of time-series information.

### ERP as a valuable feature for time-sensitive decoding

As feature vectors, we used event-related potentials (ERPs) and focused on SEPs, which are a class of ERP. In contemporary ECoG studies, the high-gamma band is the main target of analysis. This is because the high-gamma band is closely correlated with neural firing/synchrony (Ray et al., 2008) and carries rich information about certain biological signals, such as kinematics (Miller et al., 2007). In addition, high-gamma band signals cannot be easily obtained from scalp EEG.

However, to calculate band power to use such a specific band modulation (e.g., high-gamma), a time window with an appropriate length for the desired band is usually necessary. Thus, the instantaneity and accuracy of timing information may be lost, along with other limitations, according to the signal lengths used for implementing decoding algorithms (e.g., filter or buffer length). Perception and accompanying SEPs in this study reflected a very short (~several milliseconds) event, such that even event-related potentials that did not require special time windowing were considered to be useful. Indeed, we reported high accuracy (~98%) with a very short segment of data (~15 ms from stimulus onset).

ERPs are potentially suitable as feature vectors in some circumstances in which ERPs with high SNRs can be obtained by a single stimulation, like in this study. ERPs are also useful in some BMI applications in which temporal information is essential (e.g., perception of pain or hearing).

### Electrode placement toward BMI applications

According to our single-channel prediction map (Figure 6B), even within each small patch, there were some differences in prediction accuracy among the channels. In addition, as the mislocated “patch C,” which was located only about 5 mm from the optimal position showed (Figure 4A), decoding accuracy drastically decreases when the position of the array is less than ideal (Figure 6B). High-density arrays generally have small brain coverage. Thus, the optimal placement of electrode arrays is critically important to achieve high accuracy decoding with high-density arrays.

In this study, the prediction accuracy was highest in the most medial section of the arrays, and lower in the lateral section. We observed dense D3, D4, and D5 responses in the medial area, and comparably sparse D1 and D2 responses in the lateral area (Figure 5B). These tendencies suggest that, for effective decoding, the channels should be placed in an area where the activation patterns to be classified are densely represented. Unlike penetrating electrodes, it is possible to adjust the position of ECoG arrays during surgery without risking tissue damage. If it were possible to operate decoders intraoperatively during ECoG implantation, highly efficient BMIs could be developed in terms of optimization of electrode placement (decoder-suitable). Recently, Xie and colleagues recorded human ECoG during a hand motion task, performed during a craniotomy where the participant was awake (Xie et al., 2015).

The skill and precision of the neurosurgeons in this study enabled the electrodes to be partially placed into the central sulcus. Thanks to this placement, we found that single-channel prediction accuracy tended to be better in the channels in the CS (Figure 6B). In addition, the positive surface potential seen after stimulation seemed to arise within the vicinity of the CS and propagate in the direction of the postcentral gyrus (Figure 5). Sensory information ascending from the thalamus is mainly delivered to area 3 in the CS first, and subsequently reaches area 1 in the postcentral gyrus (Felleman and Van Essen, 1991). Considering the observed propagation of sensory information from the CS, it is possible that the placement of electrodes in the CS will lead to higher decoding accuracy and faster decoding capability from stimulus onset.

### Limitations and future research

Contrary to our expectations, the efficacy of high-density and multi-channel ECoG appears to be somewhat limited. One reason relates to the use of the ERP itself as a feature vector. ERPs contain signals in low-frequency bands. These low-frequency signals generally have a higher number of channel correlations, which can mask high-density effects. Another reason for the low efficacy of the high-density property of our array in our test paradigm may be our use of electrical stimulation, which is non-physiological and strong. Elicited responses were quite distinct, and prediction accuracy reached a nearly maximal value, increasing the chance that the high-density effect would be difficult to detect. To address this problem, other methods of tactile stimulation, such as a vibration motor (Hotson et al., 2016), braille device, 3-dimensional point stimulation system (Tabot et al., 2013), diaphragm-type pneumatic device (Zhu et al., 2009), or drum-type rolling device (Weber et al., 2013) may be efficacious, as they are more physiologically relevant.

Considering that others studies using different paradigms (e.g., motor-decoding) demonstrated a high-density effect, such effects may be highly dependent on stimulus type or classification/decoding tasks. Thus, we must exercise caution to avoid over-generalization of the results, and plan experiments with increasingly realistic conditions.

## Author contributions

TK and TS conceived the project. KD and TS designed and fabricated the electrode arrays. KW, KT, FY, and MH performed the surgery. TK, MY, KD, MI, HA, and TS supported the surgery and conducted the intraoperative recordings. TK analyzed the neural data. TK, KT, and TS wrote the paper.

## Funding

This work was partially supported by the Strategic Research Program for Brain Sciences by the Ministry of Education, Culture, Sports, Science and Technology of Japan, the “Research and development of technologies for high speed wireless communication from inside to outside of the body and large scale data analyses of brain information and their application for BMI” by the Commissioned Research of the National Institute of Information and Communications Technology (NICT), JSPS KAKENHI Grant Number 15H03049 and National Institute of Dental and Craniofacial Research Grant (RO1-DE023816).

### Conflict of interest statement

The authors declare that the research was conducted in the absence of any commercial or financial relationships that could be construed as a potential conflict of interest.

## Abbreviations

SEP: Sensory evoked potential
ECoG: Electrocorticogram
MEMS: Micro electro mechanical systems
COG: Center-of-gravity
BMI: Brain-machine interface
Ch: Channel
D1: Thumb
D2: Index finger
D3: Middle finger
D4: Ring finger
D5: Little finger
IPS: Intra-parietal sulcus
CS: Central sulcus
ERP: Event-related potential.
