# Supplementary material for: High Spatiotemporal Resolution ECoG Recording of Somatosensory Evoked Potentials with Flexible Micro-Electrode Arrays
Source: Front Neural Circuits. 2017 Apr 11;11:20. doi: 10.3389/fncir.2017.00020 (PMC5386975; doi:10.3389/fncir.2017.00020)
Supplement: Supplementary file 3 [file DataSheet1.docx]

Supplementary Material

High spatiotemporal resolution ECoG recording of somatosensory evoked potentials with flexible micro-electrode arrays

**Taro Kaiju^1, 2^, Keiichi Doi^1, 2^, Masashi Yokota^1, 2^, Kei Watanabe^2^, Masato Inoue^2^, Hiroshi Ando^2^, Kazutaka Takahashi^3^, Fumiaki Yoshida^2, 4^, Masayuki Hirata^2, 4^, Takafumi Suzuki^2*^**

*** Correspondence:** Takafumi Suzuki: t.suzuki@nict.go.jp

# Supplementary Data

## Supplementary Video

**Supplementary Video 1 (SEP_1mA.wmv).** Spatiotemporal dynamics of SEP elicited by stimulation with a current of 1 mA. Temporal evolution of surface potential distribution is shown. t = 0 indicates stimulation timing (*t_stim_*). Note that each patch was curved along the brain surface and one edge of the array was positioned in the central sulcus.

**Supplementary Video 2 (SEP_4mA.wmv).** Spatiotemporal dynamics of SEP elicited by stimulation with a current of 4 mA.

# Supplementary Figures and Tables

## Supplementary Tables

| **Recording Filename** | **Finger type** | **Intensity** | **Analyzed in this article** | **Timing Fix** | **Reason for exclusion** |
| --- | --- | --- | --- | --- | --- |
| Block　01 |  |  |  |  | *2 |
| Block　02 |  |  |  |  | *2 |
| Block　03 |  |  |  |  | *2 |
| Block　04 |  |  |  |  | *2 |
| Block　05 | D2 | 4 mA |  |  | *3 |
| Block　06 | D1 | 4 mA |  |  | *3 |
| Block　07 | D1 | 4 mA |  |  | *3 |
| Block　08 | D1 | 2 mA |  |  | *4 |
| Block　09 | D1 | 4 mA |  |  | Duplicated condition with Block-18 (Data is OK) |
| Block　10 | D1 | 0.5mA |  |  | *4 |
| Block　11 | D3 | 2 mA |  | *1 | *4 |
| Block　12 | D3 | 4 mA |  | *1 | Duplicated condition with Block-26 (Data is OK) |
| Block　13 | D3 | 1 mA |  |  | *4 |
| Block　14 | D4 | 1 mA | ○ |  |  |
| Block　15 | D4 | 4 mA | ○ |  |  |
| Block　16 | D5 | 4 mA | ○ |  |  |
| Block　17 | D5 | 1 mA | ○ |  |  |
| Block　18 | D1 | 1 mA | ○ |  |  |
| Block　19 | D1 | 4 mA | ○ |  |  |
| Block　20 | D2 | 4 mA | ○ | *1 |  |
| Block　21 | D2 | 1 mA | ○ |  |  |
| Block　22 | D3 | 1 mA |  |  | *5 |
| Block　23 | D3 | 4 mA |  |  | Duplicated condition with Block-26 (Data is OK) |
| Block　24 | D3 | 1 mA |  |  | *5 |
| Block　25 | D3 | 1 mA | ○ |  |  |
| Block　26 | D3 | 4 mA | ○ |  |  |

*1: For these data, the stimulus trigger was abnormally recorded. Thus, the timing information was corrected manually using the stimulus artifact.

*2: Test block for equipment setup.

*3: The stimulation interval was mistakenly set to 300 ms so that RF noise appeared even in averaged waveforms.

*4: Test block for intensity adjustment.

*5: Evoked response was absent for these trials. Stimulation artifact was also absent, indicating a technical problem with stimulation.

**Supplementary Table 1. Recording parameters and criteria for data exclusion.** An entire recorded file is shown. D1, D2, D3, D4, and D5 correspond to thumb, index, middle, ring, and pinky fingers. Duplicated data had consistent waveforms among different blocks.

## Supplementary Figures

**Supplementary Figure 1.**　Frequency response curve of the fabricated electrode. A single patch (32 channels) was measured. Gray traces show data for each channel. Red traces represent the mean of all channels (n=32). Note that this data was obtained from a different set of electrodes than those used in main study, although these electrodes had the same design and fabrication process.


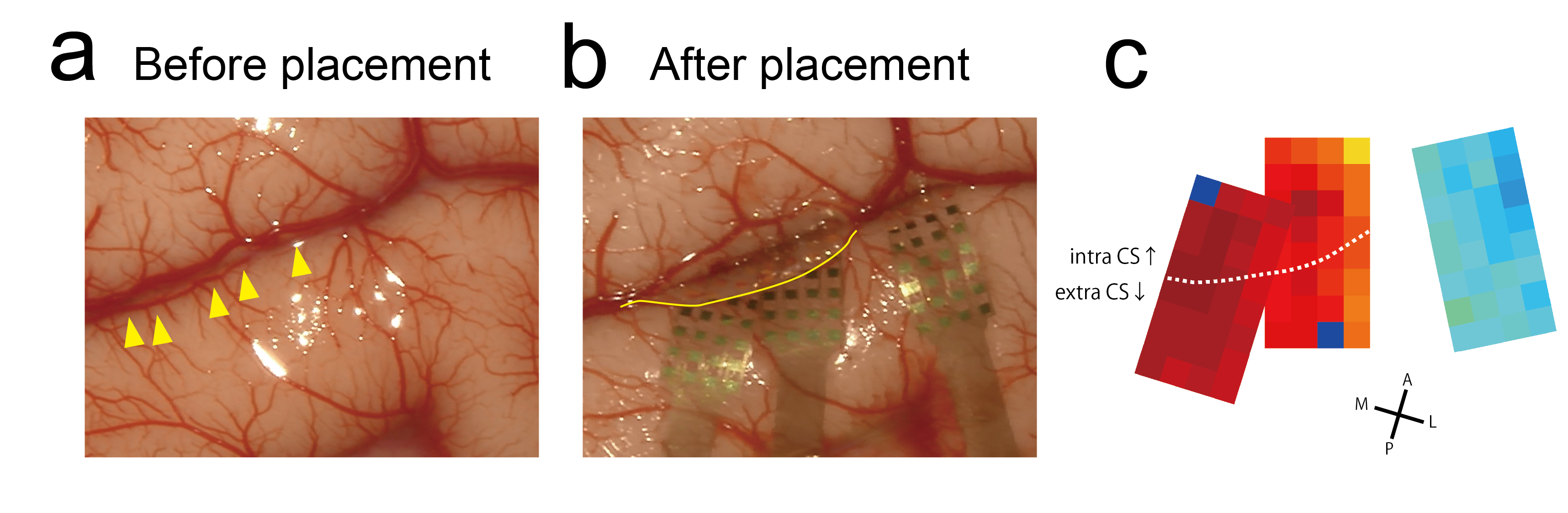
**Supplementary Figure 2.** Electrode placement at the central sulcus. **a:** Cortical surface prior to electrode placement. Yellow triangles point to a small vessel running along the central sulcus. We defined this vessel as the posterior edge of the central sulcus. **b:** Cortical surface after electrode placement. The small vessel can be seen through the transparent electrode arrays. The vessel shown in (a) is indicated by a yellow line. The most lateral array covered no suitable reference vessels near the central sulcus. **c:** According to (b), the approximate location of the small vessel is shown as a white dotted line on the accuracy map (Fig. 6b).


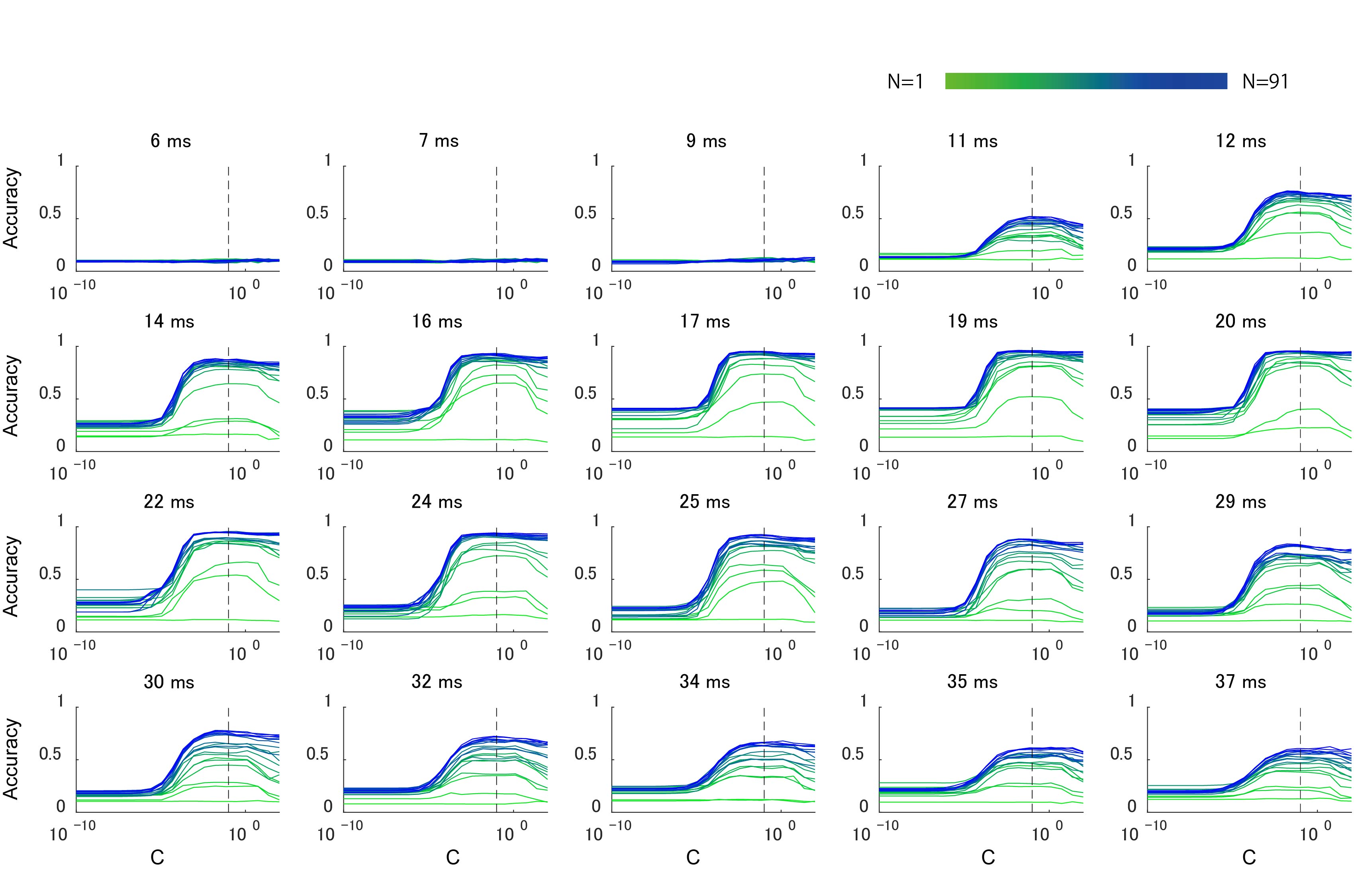


**Supplementary Figure 3.** Hyperparameter optimization for single time point prediction. Accuracy-vs-C plots corresponding to Fig. 8b are shown. The numbers above each plot are the timing at which the ECoG voltages were used in the prediction. Different traces in each plot indicate different numbers of channels (chosen via random sampling). Dotted lines indicate C = 10^-1^, which seemed to be the best option and was adopted in Fig. 8b. The indicated timings were rounded off to the nearest integer.

**
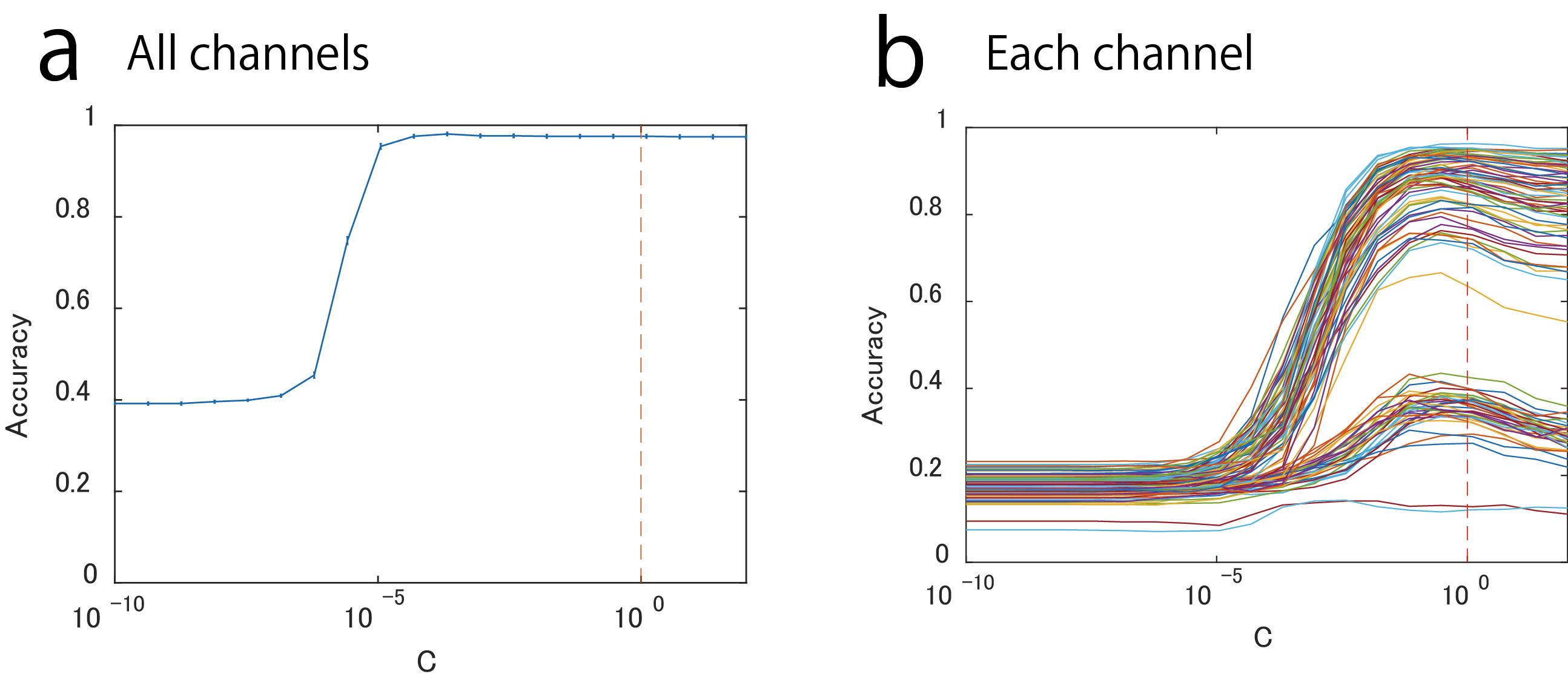
**

**Supplementary Figure 4.** **a:** Accuracy-vs-C plot for prediction using all 94 channels with a full-length sample (200 data points, about 33 ms) corresponding to Fig. 6a. A parameter space ranging from 10^-10^ to 10^2^ was divided into 20 points on a logarithmic scale. Error bars represent S.E.M. of 10-fold cross validation. The dotted line indicates C = 10^0^, which was adopted in the analysis shown in Fig. 6a. **b:** Accuracy-vs-C plot for prediction using a single channel with a full-length sample corresponding to Fig. 6b. Different traces represent different channels (Ch 1 – Ch 96, two broken channels included).


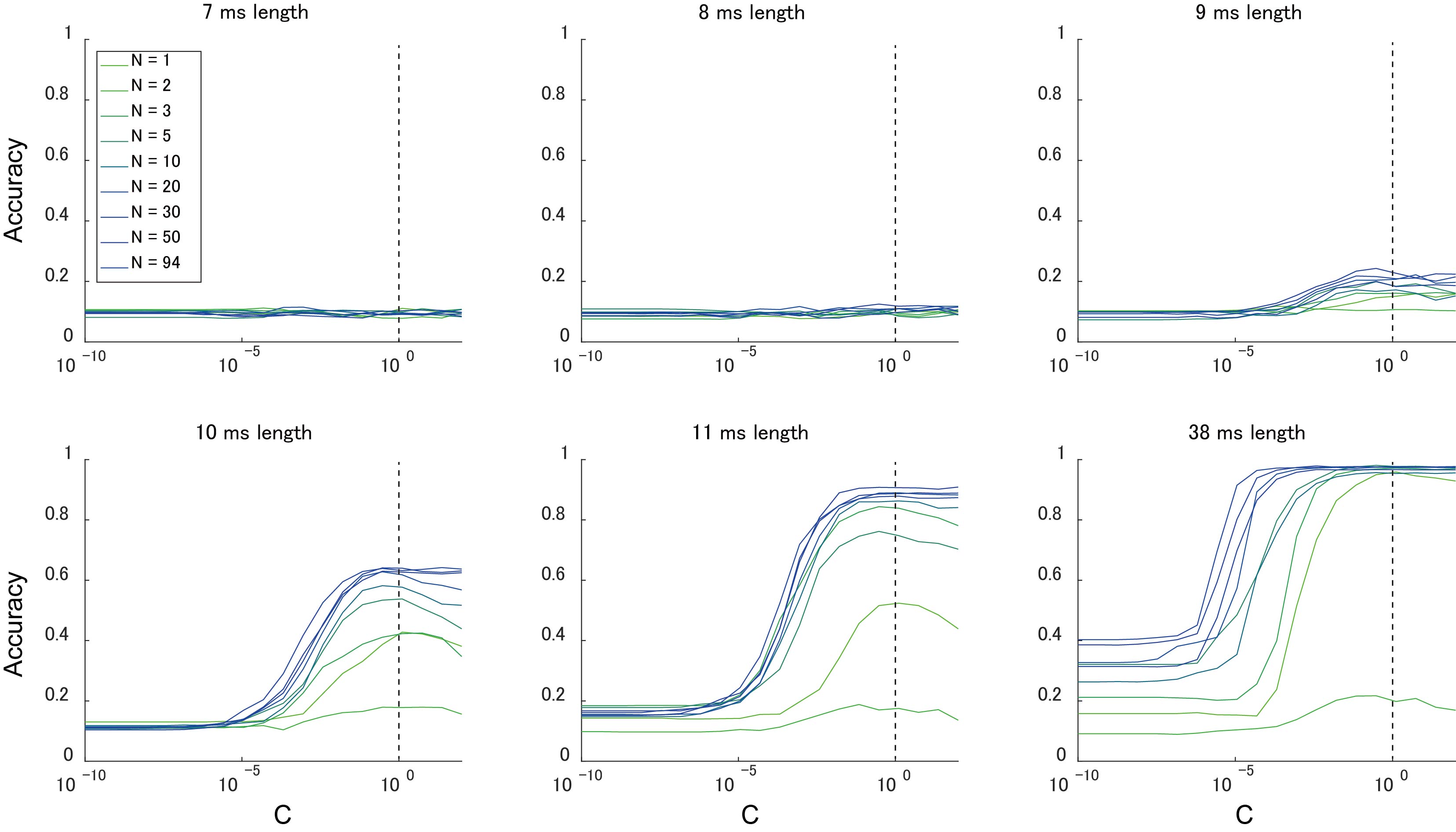


**Supplementary Figure 5.** Hyperparameter optimization for variable time-series length prediction. Accuracy-vs-C plots corresponding to Fig. 8a are shown. The length values above each plot indicate the time-series length of the ECoG data used in the prediction (length of *t_stim_* – *t_end_*, Fig.2a). Different traces in each plot indicate different numbers of channels (chosen via random sampling). Dotted lines indicate C = 10^0^, which seemed to be the best option and was adopted in the analysis shown in Fig.8a. The indicated time lengths were rounded off to the nearest integer.


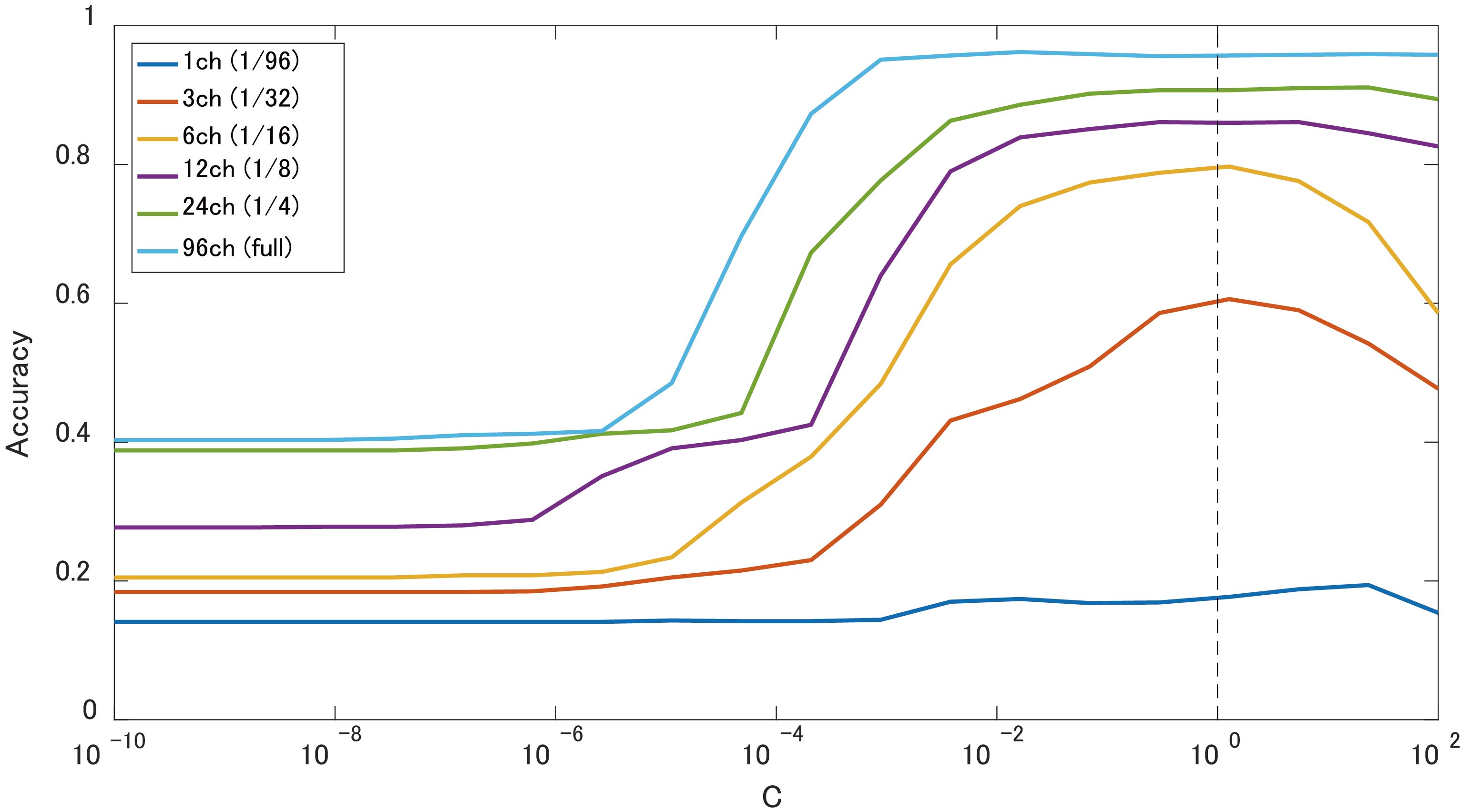


**Supplementary Figure 6.** Hyperparameter optimization for merged channel analysis. Accuracy-vs-C plots corresponding to Fig.9 are shown. Different traces indicate different numbers of channels. Dotted lines indicate C = 10^0^, which seemed to be the best option and was adopted in the analysis shown in Fig.9.
